# A high density SLAF-SNP genetic map and QTL detection for fibre quality traits in *Gossypium hirsutum*

**DOI:** 10.1186/s12864-018-5294-5

**Published:** 2018-12-06

**Authors:** Iftikhar Ali, Zhonghua Teng, Yuting Bai, Qing Yang, Yongshui Hao, Juan Hou, Yongbin Jia, Lixia Tian, Xueying Liu, Zhaoyun Tan, Wenwen Wang, Kiirya Kenneth, Abdalla Yousef Ahmed Sharkh, Dexin Liu, Kai Guo, Jian Zhang, Dajun Liu, Zhengsheng Zhang

**Affiliations:** College of Agronomy and Biotechnology, Southwest University, Chongqing, 400716 China

**Keywords:** Upland cotton (*Gossypium hirsutum* L.), Genetic map, Fiber quality traits, Quantitative trait loci mapping, Specific locus amplified fragment sequencing (SLAF-seq), Single nucleotide polymorphism marker

## Abstract

**Background:**

Upland Cotton (*Gossypium hirsutum*) is a very important cash crop known for its high quality natural fiber. Recent advances in sequencing technologies provide powerful tools with which to explore the cotton genome for single nucleotide polymorphism marker identification and high density genetic map construction toward more reliable quantitative trait locus mapping.

**Results:**

In the present study, a RIL population was developed by crossing a Chinese high fiber quality cultivar (Yumian 1) and an American high fiber quality line (CA3084), with distinct genetic backgrounds. Specific locus amplified fragment sequencing (SLAF-seq) technology was used to discover SNPs, and a genetic map containing 6254 SNPs was constructed, covering 3141.72 cM with an average distance of 0.5 cM between markers. A total of 95 QTL were detected for fiber quality traits in three environments, explaining 5.5-24.6% of the phenotypic variance. Fifty-five QTL found in multiple environments were considered stable QTL. Nine of the stable QTL were found in all three environments. We identified 14 QTL clusters on 13 chromosomes, each containing one or more stable QTL.

**Conclusion:**

A high-density genetic map of *Gossypium hirsutum* developed by using specific locus amplified fragment sequencing technology provides detailed mapping of fiber quality QTL, and identification of ‘stable QTL’ found in multiple environments. A marker-rich genetic map provides a foundation for fine mapping, candidate gene identification and marker-assisted selection of favorable alleles at stable QTL in breeding programs.

**Electronic supplementary material:**

The online version of this article (10.1186/s12864-018-5294-5) contains supplementary material, which is available to authorized users.

## Background

Cotton, the main source of natural textile fiber, is one of the most important cash crops. Cultivated species of cotton include two diploids (*Gossypium herbaceum* and *G. arboreum)* and two tetraploids (*G. hirsutum* and *G. barbadense).* Due to its higher yield and wide adaptability, *G. hirsutum* L. accounts for more than 95% of worldwide cotton production [1]. Cotton fiber yield is of primary importance for cotton growers, whereas the textile industry demands high fiber quality. In order to meet the diverse demands of cotton growers and the textile industry, it is necessary for cotton breeders to develop cultivars with both high yield and superior fiber quality. Cotton fiber quality components, important for spinning, primarily include length, strength and micronaire (a measure of fineness). These traits have genetically complex quantitative inheritance and are substantially influenced by environmental factors. Traditional breeding approaches are often not precise enough to uncover the genetic architecture of these traits. Marker assisted selection (MAS) has proven to be a robust and cost effective breeding tool to manipulate genes controlling quantitative traits [2, 3].

In the past few decades, the efficiency of QTL mapping has been improved with the advent of molecular markers. More than 1500 QTL for fiber quality traits have been reported [4]. Simple sequence repeats (SSR), a type of DNA marker, have been used for genetic mapping and QTL detection for about two decades. However, low polymorphism rate limits the use of SSRs for creating high density cotton genetic maps. Recent advances in ‘next generation sequencing (NGS)’ enabled researchers to identify DNA sequence polymorphisms i.e. SNPs, scanning large numbers of nucleotides to find sufficiently large subsets for genome mapping even between closely-related parents. Therefore, SNP markers are effective for creating high density genetic maps, mapping QTL and use in MAS [5–8], understanding population structure and studying genetic diversity [9].

The release of genome sequences of cotton species including *G. raimondii* [10, 11], *G. arboreum* [12], *G. hirsutum* [13, 14], and *G. barbadense* [15] made it easy to utilize NGS for construction of high density genetic maps and QTL identification. A variety of technologies were developed based on NGS, such as genotyping by sequencing (GBS) [16], restriction-site associated DNA sequencing (RAD-seq) [17] and specific locus amplified fragment sequencing (SLAF-seq) [18]. SLAF-seq is one of the most efficient and cost effective tools for manipulating SNPs and genotyping [18]. SLAF-seq has many merits over the other advanced technologies: 1) it does not necessarily require a reference genome sequence and polymorphism information; 2) repetitive sequences can be avoided; and 3) a balance between marker density and population size can be maintained by varying the fragment size. SLAF-seq has previously been applied in many plant species for developing high density genetic maps and mapping QTL [19–23]. Shen et al. [23] identified 132,880 SNPs and 6,296 InDels between a reference genome (TM-1) and five tetraploid cotton species by using SLAF-seq. Zhang et al. [24] exploited SLAF-seq to construct a genetic map of 5521 SNPs covering 3259.4 cM and reveal 18 QTL for cotton boll weight, explaining 4.15-16.70% phenotypic variance, with 344 putative candidate genes identified.

In this study, a RIL population of 180 lines from a cross between a Chinese high fiber quality cultivar (Yumian 1) and an American high fiber quality line (CA3084) was investigated, with these parents selected based on their distinct genetic background. SLAF-seq was used for the discovery of SNPs, which were then used to develop a high density genetic map and identify QTL for fiber quality traits.

## Results

### Phenotypic analysis of fiber quality traits

Descriptive statistics for fiber quality traits i.e. fiber length (FL), fiber uniformity (FU), fiber micronaire (FM), fiber elongation (FE) and fiber strength (FS) across three environments were summarized in Table 1. In all environments, the fiber length and strength of CA3084 were higher than Yumian 1. All five traits changed according to circumstances for both parents and population. Besides, the phenotype of RIL population showed continuous variation and transgressive segregation. Skewness and kurtosis tests showed that all these traits were normally distributed (Table 1).

**Table 1:** Phenotypic variation of fiber quality traits for Yumian1, CA3084, and their RIL population

Trait^a^	Env^b^	Parents			RIL Population					
		P1^c^	P2^d^	P2-P1	Mean	Min	Max	Skew.	Kurt.	SD
FL	2015-CQ	29.5	32.7	3.2	30.1	26.8	33.9	0.2	0.5	1.3
	2016-CQ	29.1	33.3	4.2	30.4	26.4	34.1	0.3	-0.1	1.7
	2016-HN	29.9	32.0	2.1	29.9	26.2	33.6	0.0	0.1	1.4
FU	2015-CQ	85.2	85.0	-0.2	84.8	82.2	87.2	0.1	-0.3	1.1
	2016-CQ	84.8	85.8	1.0	86.1	81.5	88.6	-0.7	2.0	1.1
	2016-HN	85.5	84.1	-1.4	83.5	79.1	86.8	-0.1	-0.1	1.5
FM	2015-CQ	3.9	4.1	0.2	3.9	3.1	5.0	0.6	0.8	0.3
	2016-CQ	4.8	4.4	-0.40	4.3	3.5	5.4	0.4	-0.2	0.4
	2016-HN	3.0	3.7	0.7	3.5	2.6	4.7	0.6	0.9	0.4
FE	2015-CQ	6.8	7.0	0.2	6.7	6.6	7.0	0.1	0.0	0.1
	2016-CQ	6.8	7.2	0.4	6.8	6.5	7.1	0.0	-0.2	0.1
	2016-HN	6.7	6.8	0.1	6.7	6.5	6.9	0.2	-0.4	0.1
FS	2015-CQ	32.6	38.2	5.6	33.3	28.2	40.7	0.3	0.6	2.3
	2016-CQ	35.8	42.0	6.2	36.3	28.3	44.4	0.0	0.2	3.4
	2016-HN	29.3	34.4	5.1	30.3	25.8	36.9	0.2	-0.1	2.1

^a^FL: fiber length; FU: fiber uniformity; FS: fibers strength; FM: fiber micronaire; FE: fiber elongation.^b^ 2015CQ, 2015 in Chongqing; 2016CQ, 2016 in Chongqing; 2016HN, 2016 in Hainan. ^c^ parent Yumian 1.^d^ parent CA3084.

One-way ANOVA showed that all traits had significant genetic and environmental effects (*p* < 0.01) (Table 2). Correlations among all five fiber quality traits were significant except FU-FM and FM-FE (Table 3).

**Table 2:** Analysis of variance (ANOVA) for fiber quality traits in the Yumian 1 × CA3084 RIL population across three environments

Trait^a^	Source	*df*	Mean square	*F* value
FL	Genotype	179	4.85	7.88^**^
	Environment	2	11.49	18.67^**^
	Error	358	0.62	
FU	Genotype	179	3.24	4.28^**^
	Environment	2	314.66	416.21^**^
	Error	358	0.76	
FS	Genotype	179	14.93	6.07^**^
	Environment	2	1577.97	641.51^**^
	Error	358	2.46	
FM	Genotype	179	0.29	5.51^**^
	Environment	2	29.21	556.59^**^
	Error	358	0.05	
FE	Genotype	179	0.02	4..23^**^
	Environment	2	0.46	82.46^**^
	Error	358	0.01	

^a^FL fiber length; FU fiber uniformity, FM fiber micronaire, FE fiber elongation, FS fiber strength

*, ** Significant differences with a probability level of 0.05 and 0.01, respectively. Error reflects variation caused by factors other than genotype or environment.

**Table 3:** Correlation coefficients among fiber quality traits in the Yumian 1 × CA3084 RIL population across three environments

Traits^a^	FL	FU	FS	FM	FE
FL	1				
FU	0.363^**^	1			
FS	0.759^**^	0.505^**^	1		
FM	-0.356^**^	0.063	-0.217^*^	1	
FE	0.808^**^	0.439^**^	0.822^**^	-0.089	1

^a^FL fiber length; FU fiber uniformity, FM fiber micronaire, FE fiber elongation, FS fiber strength

*, ** Significant differences with a probability level of 0.05 and 0.01 respectively

### Analysis of SLAF-seq data and SLAF markers

After SLAF library construction and high-throughput sequencing, a total of 83.65 GB data comprising 418.26 M paired-end reads was generated. Among them, 93.80% of bases met or exceeded a quality score of 30 (i.e., Q30, indicating a 0.1 % chance of an error, and thus 99.9% confidence) and guanine-cytosine (GC) content was 38.54%. The 418.26 M paired-end reads consisted of 158985 SNP markers. A total of 33288 (20.93% of) markers were polymorphic in the RIL population. The polymorphic SNP markers were classified into four genotypes as follows: aa × bb meant that both parents were homozygous in this SNP position, the genotype of one parent was aa and the other was bb; hk × hk meant that both parents were heterozygous; and the lm × ll and nn × np meant that one parent was heterozygous and the other was homozygous. Only genotype aa × bb, consisting of 21781 SNPs, was used for further analysis in our study. Markers with low sequencing depth or more than 30% missing data were filtered out. A total of 6254 markers meeting these quality standards were used for genetic map construction.

### Construction of the genetic map

**Fig. 1:**
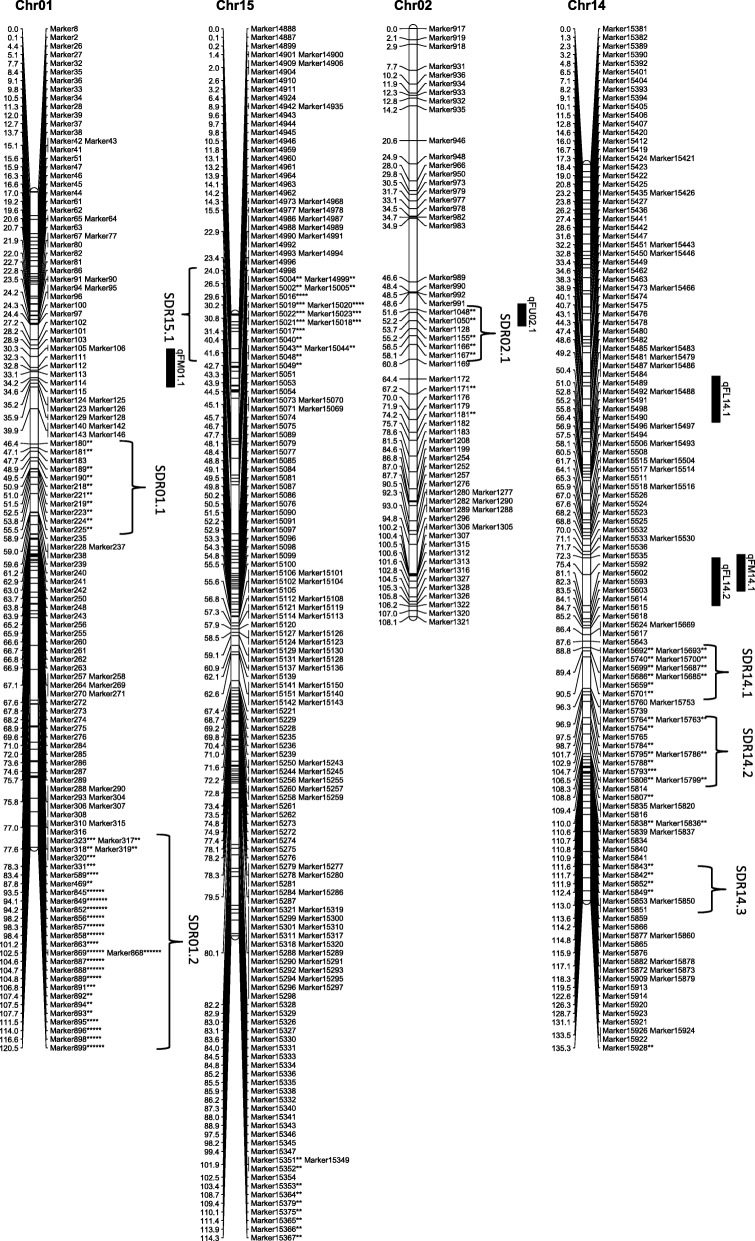
Genetic maps of Chr01/Chr15 and Chr02/Chr14 homoeologous chromosomes and QTL for fiber quality in the Yumian 1 × CA3084 RIL population. Map distances were given in centiMorgans (cM). Markers favoring Yumian 1 alleles were followed by asterisks with the level of ** *P* < 0.05, *** *P* < 0.01, **** *P* < 0.005, ***** *P* < 0.001, ****** *P* < 0.0005, ******* *P* < 0.0001 according the standard of Joinmap 4.0. The replaced sign (#) indicated markers favoring CA3084 alleles. Bars along the genetic map indicated the QTL likelihood interval. QTL were shown as FL for fiber length, FU for fiber uniformity, FS for fiber strength, FE for fiber elongation, and FM for fiber micronaire

**Fig. 2:**
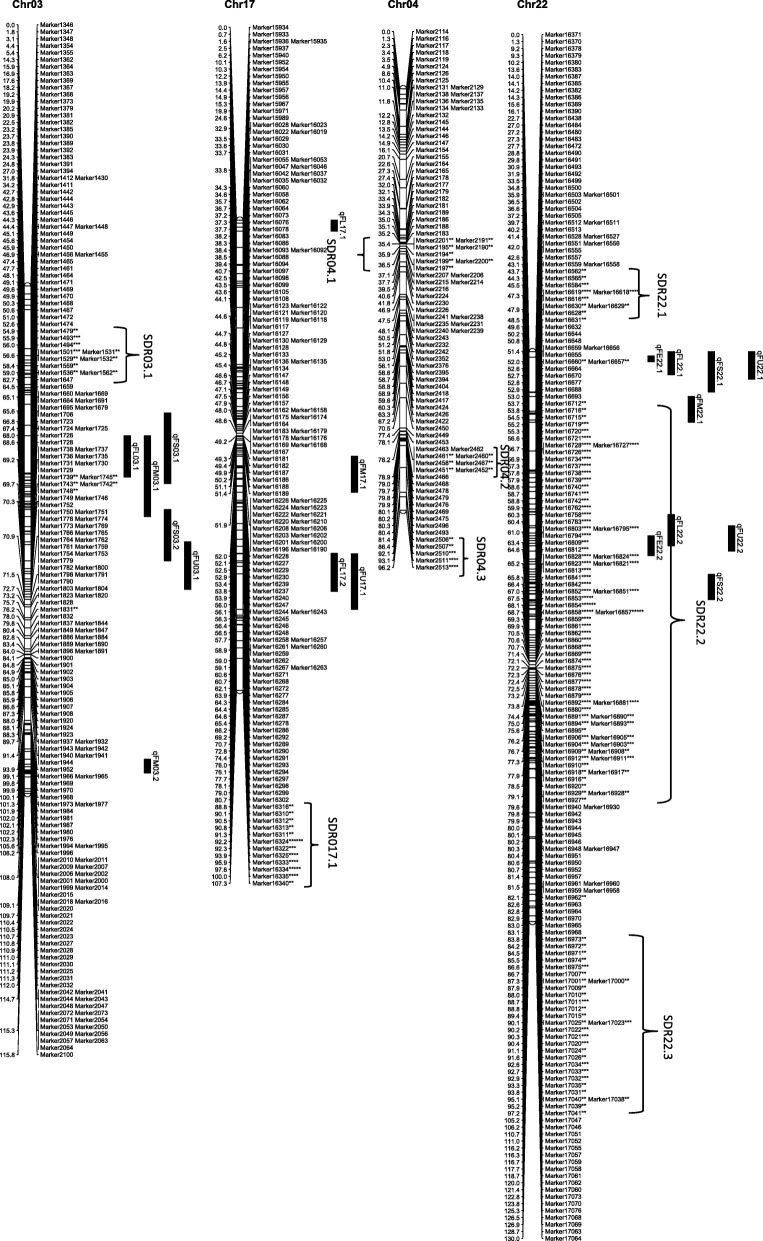
Genetic maps of Chr03/Chr17 and Chr04/Chr22 homoeologous chromosomes and QTL for fiber quality in the Yumian 1 × CA3084 RIL population. All notes are the same as Fig. 1

**Fig. 3:**
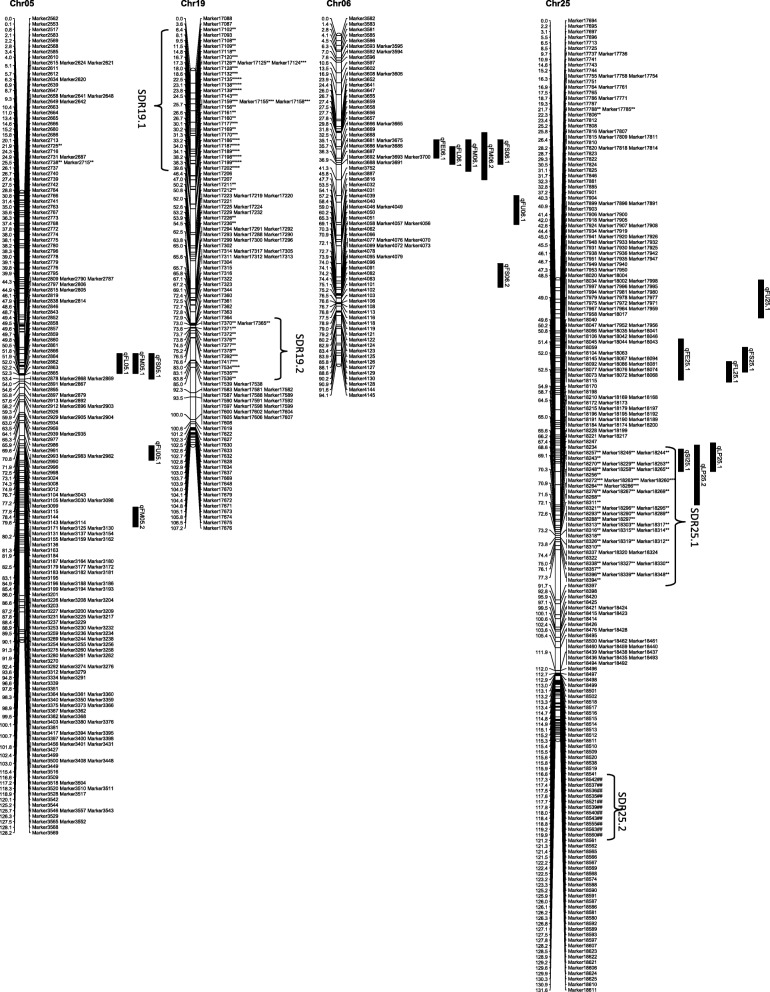
Genetic maps of Chr05/Chr19 and Chr06/Chr25 homoeologous chromosomes and QTL for fiber quality in the Yumian 1 × CA3084 RIL population. All notes are the same as Fig. 1

**Fig. 4:**
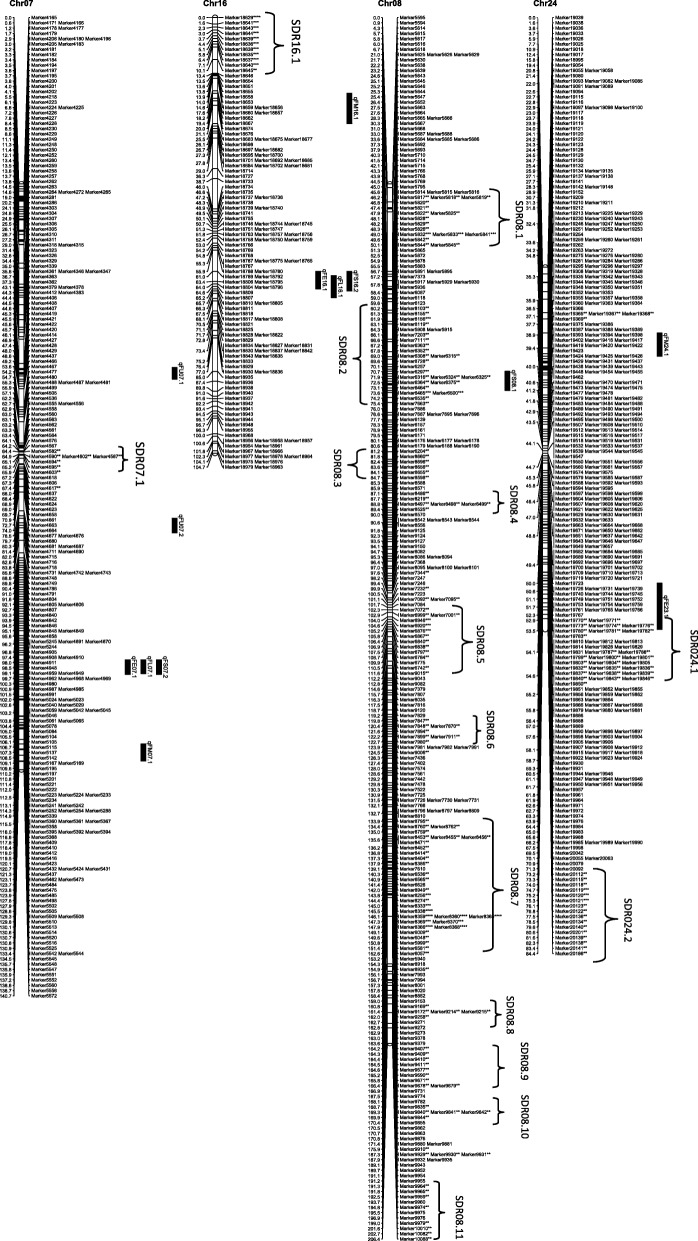
Genetic maps of Chr07/Chr16 and Chr08/Chr24 homoeologous chromosomes and QTL for fiber quality in the Yumian 1 × CA3084 RIL population. All notes are the same as Fig. 1

**Fig. 5:**
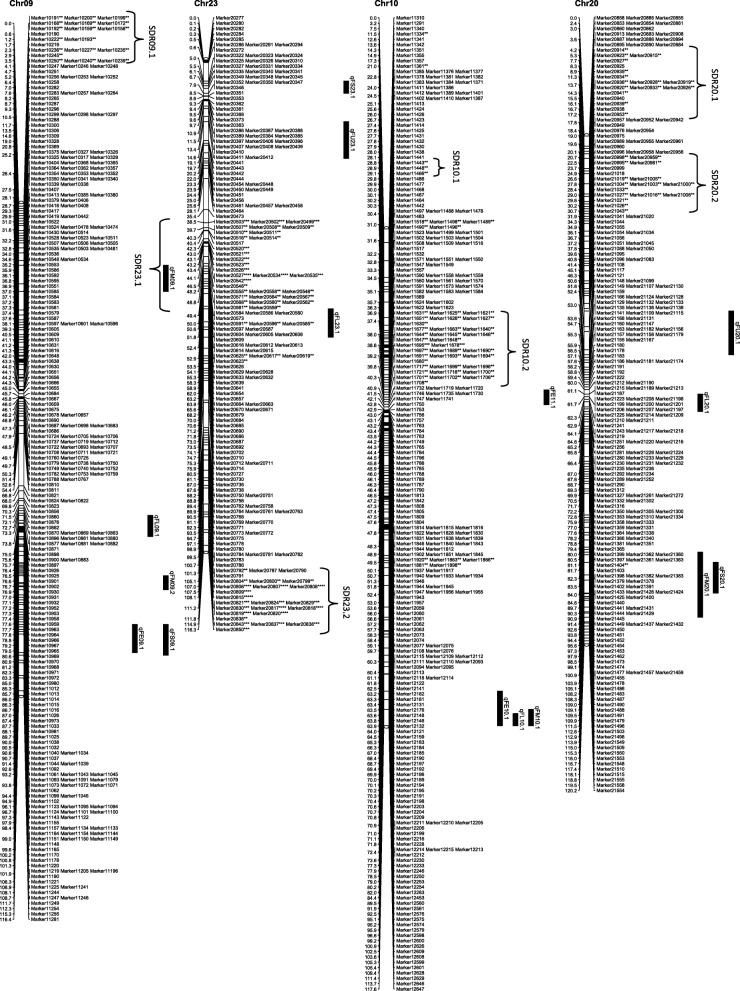
Genetic maps of Chr09/Chr23 and Chr10/Chr20 homoeologous chromosomes and QTL for fiber quality in the Yumian 1 × CA3084 RIL population. All notes are the same as Fig. 1

**Fig. 6:**
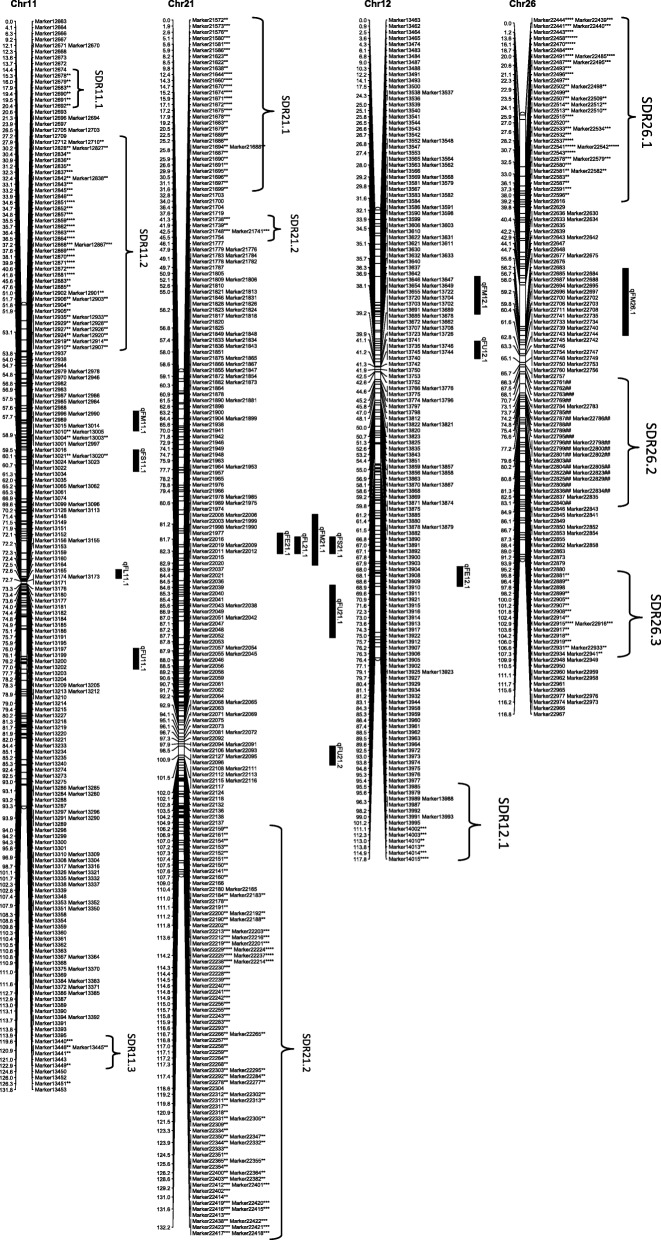
Genetic maps of Chr11/Chr21 and Chr12/Chr26 homoeologous chromosomes and QTL for fiber quality in the Yumian 1 × CA3084 RIL population. All notes are the same as Fig. 1

**Fig. 7:**
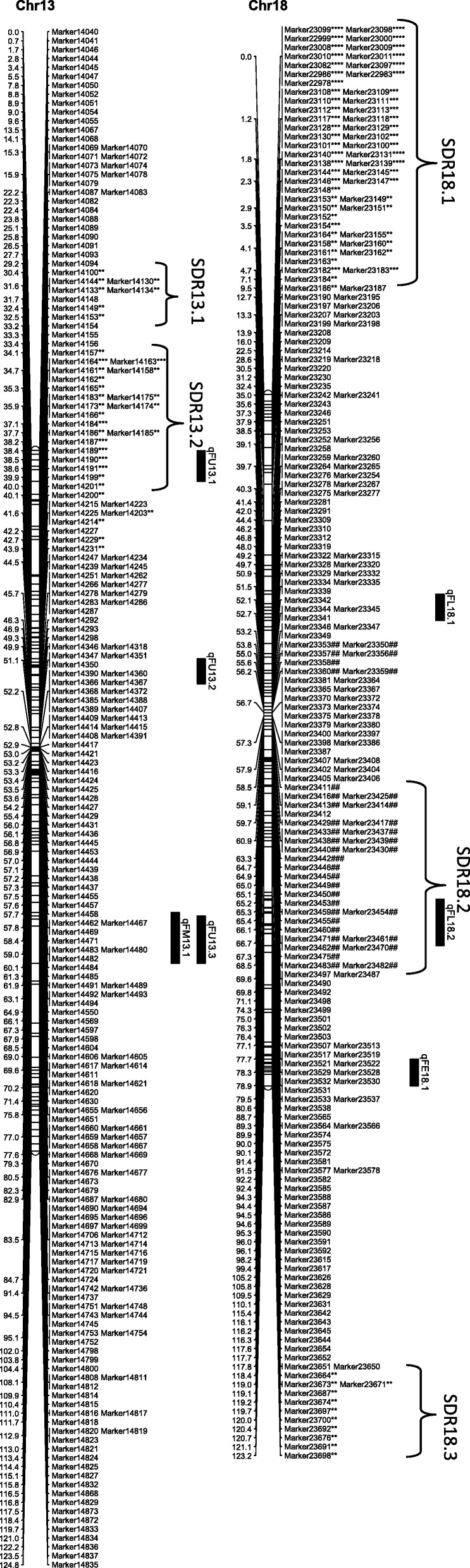
Genetic maps of Chr13/Chr18 homoeologous chromosomes and QTL for fiber quality in the Yumian 1 × CA3084 RIL population. All notes are the same as Fig. 1

From the 6254 high quality SNP markers, a genetic map covering a total distance of 3141.7 cM with an average marker interval of 0.5 cM was constructed (Table 4; Figs. 1, 2, 3, 4, 5, 6 and 7). The A sub-genome harbored 3430 markers covering 1618.4 cM whereas the D sub-genome harbored 2824 markers covering 1523.3 cM. The largest chromosome was Chr08, containing 958 markers covering 206.4 cM, with an average marker interval of 0.2 cM. The shortest chromosome was Chr06, containing 86 markers covering 94.1 cM with an average marker interval of 1.1 cM. There were only 8 gaps greater than 10.0 cM, the largest being 14.353 cM on Chr08 (Table 4).

**Table 4:** Marker distribution and chromosome parameters on the enriched genetic map of the Yumian 1 × CA3084 RIL population

Chr	Loci	Genetic Distance (cM)	Average interval	largest gap (cM)	No. gaps (> 10 cM)	Physical Length (Mb)	Genome Coverage (%) ^a^	SDMs	SDMs (%)	SDRs
Chr01	141	120.5	0.9	6.6	0	99.3	99.4	38	27.0	2
Chr02	63	108.1	1.7	11.6	1	79.4	95.2	7	11.1	1
Chr03	201	115.9	0.6	8.9	0	95.7	95.5	16	8.0	1
Chr04	94	96.2	1.0	6.9	0	61.6	98.0	19	20.2	3
Chr05	241	128.2	0.5	12.4	1	89.5	97.3	3	1.2	0
Chr06	86	94.1	1.1	7.0	0	100.6	97.5	0	0.0	0
Chr07	465	140.7	0.3	5.8	0	72.3	92.4	11	2.4	1
Chr08	958	206.4	0.2	14.4	2	91.3	88.1	506	52.8	11
Chr09	269	116.4	0.4	12.4	1	73.0	97.4	18	6.7	1
Chr10	274	117.6	0.4	5.1	0	99.3	98.4	49	17.9	2
Chr11	230	131.7	0.6	7.1	0	90.5	97.0	62	27.0	3
Chr12	176	117.8	0.7	10.0	0	86.2	98.6	6	3.4	1
Chr13	232	124.8	0.5	6.9	0	76.4	95.6	33	14.2	2
**A** ^**t**^	**3430**	**1618.4**	**0.5**	**114.9**	**5**	**1115.2**	**96.2**	**768**	**22.4**	**28**
Chr14	152	135.3	0.9	5.8	0	66.8	99.2	28	18.4	3
Chr15	190	114.3	0.6	9.0	0	60.3	98.1	26	13.7	2
Chr16	128	104.7	0.8	8.1	0	52.7	95.2	11	8.6	1
Chr17	154	107.3	0.7	8.3	0	46.0	98.6	12	7.8	1
Chr18	238	123.2	0.5	8.1	0	60.4	99.7	101	42.4	3
Chr19	125	107.2	0.9	8.0	0	60.2	97.3	45	36.0	2
Chr20	254	120.2	0.5	5.1	0	60.1	94.8	32	12.6	2
Chr21	272	132.2	0.5	4.4	0	62.9	95.2	126	46.3	3
Chr22	290	130.0	0.5	8.0	0	46.2	89.7	160	55.2	3
Chr23	195	116.2	0.6	7.4	0	49.7	97.4	60	30.8	2
Chr24	365	84.4	0.2	3.6	0	61.7	93.6	46	12.6	2
Chr25	290	131.6	0.5	14.4	1	63.2	98.4	62	21.4	2
Chr26	171	116.8	0.7	12.4	2	58.0	98.1	84	49.1	3
**D** ^**t**^	**2824**	**1523.3**	**0.5**	**102.5**	**3**	**748.0**	**96.6**	**793**	**28.1**	**29**
**Total**	**6254**	**3141.7**	**0.5**	**217.4**	**8**	**1863.2**	**96.4**	**1561**	**25.0**	**57**

*Chr* chromosome, *SDM* Segregation distorted Marker, *SDR* segregation distorted region

^a^Percentage of pseudomolecules assembled by Zhang et al [13] spanned by the genetic map of the indicated chromosome

The boldfaced entries represent the subtotal for each subgenome of *Gossypium hirsutum*

### Segregation distortion

Among 6254 mapped loci, 1561 (24.96%) showed segregation ratios that deviated significantly (*p* <0.05) from Mendelian expectations (Table 4). These segregation distortion markers (SDMs) were unevenly distributed over the genome, forming 56 segregation distortion regions (SDRs). A total of 768 SDMs in 28 SDRs were located on the A subgenome and 793 SDMs in 29 SDRs were located on the D subgenome. A remarkable 52.8% (506) of the total markers on Chr08 had significant segregation distortion, while Chr06 had no SDMs and Chr05 had only 3 SDMs (Table 4; Figs. 1, 2, 3, 4, 5, 6 and 7). A total of 1486 SDMs (95.19%) favored Yumian 1 alleles while 75 SDMs (4.81%) favored CA3084.

### Collinearity between the genetic and the physical map

**Fig. 8:**
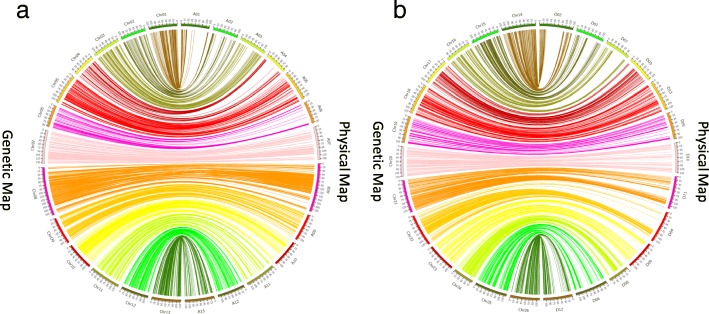
Colinearity between the Yumian 1 × CA3084 genetic map and physical map. **a** Colinearity for the A^t^ 581 subgenome. **b** Colinearity for the D^t^ subgenome

Collinearity with the physical map was studied to assess the quality of the genetic map. The physical maps of 26 chromosomes were constructed based on the positions of 6254 mapped loci on the reference genome sequence of *G. hirsutum* [13], and covered 96.53% of the genome. All chromosomes showed more than 90% coverage except Chr08 and Chr22 which showed 88.08% and 89.97% coverage, respectively (Additional file 1: Table S1). Most genetically mapped loci were collinear with their physical map locations (Fig. 8).

### QTL mapping

A total of 95 QTL for fiber quality traits were identified in this study (Additional file 2: Table S2; Figs. 1, 2, 3, 4, 5, 6 and 7). Phenotypic variance explained (PVE) by these QTL ranged from 5.5-24.6% and LOD values ranged from 2.0-10.6. The A^t^ genome contained 50 QTL, while the D^t^ genome contained 45 QTL. Every chromosome had at least one QTL except Ch04, Ch15, and Ch19. Among the 95 detected QTL, Yumian 1 contributed 46 favorable alleles while CA3084 contributed 49. A total of 55 QTL were found in more than one environment with 9 found in all three environments (Table 5).

**Table 5:** Stable quantitative trait loci (QTL) for fiber quality and yield component traits identified in the Yumian 1 × CA3084 RIL population

Trait^a^	QTL Name	Chr	Position	Nearest Marker	LOD	Additive^b^	PVE^c^(%)
FE	qFE06.1	6	32.50	Marker3668	4.2	0.03	11.6
	qFE09.1	9	101.35	Marker11220	3.0	-0.04	8.2
	qFE10.1	10	111.37	Marker12629	3.5	-0.05	9.7
	qFE11.1	11	39.91	Marker12871	3.4	-0.03	9.4
	qFE12.1	12	96.25	Marker13988	2.9	-0.03	8.2
	qFE16.1	16	63.41	Marker18806	3.0	0.03	8.3
	qFE21.1	21	46.14	Marker21777	4.8	0.03	12.0
	qFE25.1	25	23.43	Marker17812	10.6	-0.04	24.6
FL	qFL06.1	6	35.14	Marker3681	4.0	0.45	11.1
	qFL07.1	7	114.88	Marker5300	6.0	0.66	15.9
	qFL09.1	9	75.59	Marker10900	2.3	-0.34	6.4
	qFL10.1	10	117.62	Marker12647	8.9	-0.81	22.7
	qFL11.1	11	70.19	Marker13126	3.5	-0.52	9.6
	qFL22.1	22	1.27	Marker16370	3.6	0.45	9.2
	qFL25.1	25	28.17	Marker17818	3.6	-0.44	9.3
	qFL16.1	16	63.41	Marker18806	2.4	0.34	6.6
	qFL23.1	23	63.19	Marker20657	3.8	-0.43	10.5
	qFL21.1	21	46.14	Marker21777	6.9	0.58	18.1
	qFL18.1	18	38.51	Marker23253	3.1	-0.39	8.5
	qFL18.2	18	95.30	Marker23590	2.8	-0.38	7.9
FM	qFM03.2	3	109.14	Marker2016	4.5	0.14	12.1
	qFM05.1	5	49.48	Marker2858	4.4	-0.14	11.9
	qFM05.2	5	91.28	Marker3260	3.3	0.11	8.5
	qFM07.1	7	135.94	Marker5551	6.7	-0.17	17.4
	qFM09.2	9	90.05	Marker11038	2.7	-0.09	7.6
	qFM11.1	11	30.44	Marker12834	2.8	0.09	7.9
	qFM12.1	12	25.00	Marker13539	4.0	0.11	11.0
	qFM13.1	13	83.48	Marker14712	4.9	-0.15	13.2
	qFM14.1	14	75.35	Marker15592	2.9	0.09	8.0
	qFM16.1	16	19.95	Marker18674	3.2	-0.12	8.7
	qFM20.1	20	100.87	Marker21457	3.4	0.13	9.5
	qFM21.1	21	0.00	Marker21572	4.7	-0.14	11.9
	qFM26.1	26	42.25	Marker22639	2.9	0.11	7.6
FS	qFS03.1	3	31.80	Marker1430	3.3	0.71	9.2
	qFS06.1	6	32.50	Marker3668	6.8	1.43	17.7
	qFS07.1	7	114.88	Marker5313	2.3	0.60	6.6
	qFS09.1	9	100.18	Marker11170	2.8	-0.94	7.7
	qFS10.1	10	117.62	Marker12647	5.2	-1.29	13.8
	qFS12.2	12	94.84	Marker13975	4.3	-0.82	11.9
	qFS16.2	16	63.41	Marker18806	2.5	0.62	7.0
	qFS18.1	18	38.51	Marker23253	2.8	-0.03	7.9
	qFS20.1	20	105.10	Marker21486	2.8	-0.93	7.7
	qFS21.1	21	46.14	Marker21777	2.9	0.68	8.2
	qFS22.1	22	1.27	Marker16370	5.5	0.81	13.6
FU	qFU02.1	2	52.21	Marker1050	2.3	0.28	6.5
	qFU03.1	3	65.07	Marker1691	3.2	0.32	8.9
	qFU05.1	5	74.29	Marker3008	5.1	-0.55	12.7
	qFU06.1	6	47.65	Marker3816	4.2	0.36	11.5
	qFU07.1	7	45.57	Marker4422	3.3	0.32	9.2
	qFU12.1	12	35.14	Marker13621	3.2	0.32	8.9
	qFU13.1	13	0.69	Marker14041	6.4	-0.63	15.7
	qFU17.1	17	78.97	Marker16299	2.2	0.27	6.3
	qFU25.1	25	2.21	Marker17695	5.1	-0.44	13.7
	qFU23.1	23	22.04	Marker20444	3.5	0.33	9.7
	qFU21.1	21	62.65	Marker21898	2.5	-0.28	6.9

^a^FL: fiber length, FU: fiber uniformity, FS: fiber strength, FE: fiber elongation, FM: fiber micronaire. ^b^ Positive additive effects indicated that Yumian 1 alleles increased the phenotypic value, negative additive effects suggested that CA3084 alleles increased the phenotypic value. ^c^ phenotypic variance explained.

### Fiber Length

Twenty QTL controlling fiber length were found on 16 chromosomes, explaining 5.7-22.7% of the phenotypic variance (Additional file 2: Table S2). Ch14, Ch17, Ch18 and Ch22 each had 2 fiber length QTL. Yumian1 contributed 8 favorable alleles whereas CA3084 contributed 12. Among all fiber QTL, 12 were found in two or more environments, but only 3 (qFL06.1, qFL21.1, qFL23.1) were found in all three (Table 5).

### Fiber Strength

Twenty-one fiber strength QTL were found on 16 chromosomes, explaining 6-17.7% of the phenotypic variance (Additional file 2: Table S2). Ch03, Ch06, Ch16 and Ch22 each had 2 fiber strength QTL. Yumian1 contributed 10 favorable alleles for fiber strength whereas CA3084 contributed 11. Eleven QTL were detected in two or more environments, but only qFS12.2 was detected in all three. Five fiber strength QTL (qFS03.2, qFS06.1, qFS12.2, qFS22.1, qFS23.1) explained more than of 10% phenotypic variance (Table 5).

### Fiber Micronaire

Twenty-two QTL controlling fiber micronaire were found on 18 chromosomes, explaining 5.5-17.4% of the phenotypic variance (Additional file 2: Table S2). Ch03, Ch05, Ch06 and Ch09 each had two QTL. Yumian1 contributed 10 favorable alleles for fiber micronaire while CA3084 contributed 12. Thirteen QTL were found in two or more environments, and three (qFM07.1, qFM12.1, qFM21.1) in all three (Table 5). Three QTL (qFM03.2, qFM12.1, qFM13.1) explained more than 10% of the phenotypic variance.

### Fiber Elongation

Thirteen fiber elongation QTL were found on 12 chromosomes, explaining 6.1-12% of the phenotypic variance (Additional file 2: Table S2). Only two QTL (qFE06.1, qFE25.1) explained more than 10% phenotypic variance. CA3084 contributed 8 favorable alleles, while Yumian1 contributed 5. Eight QTL were found in two or more than two environments, but only qFE16.1 was found in all three, explaining 8.3% of the phenotypic variance (Table 5).

### Fiber Uniformity

Nineteen QTL controlling fiber uniformity were detected on 15 chromosomes, explaining 5.6-15.7% of the phenotypic variance (Additional file 2: Table S2). Three QTL (qFU06.1, qFU20.1, qFU21.2) explained more than 10% phenotypic variance. Yumian1 contributed 8 favorable alleles for fiber uniformity, while CA3084 contributed 11. Eleven QTL were found in two or more than two environments, but only qFU12.1 was detected in all three (Table 5).

### Cluster analysis

QTL clusters were defined as regions which contained multiple QTL associated with different traits within approximately 20 cM [25]. In this study 14 QTL clusters were found on 13 chromosomes (Table 6). The details of clusters have been provided in the supplementary files (Additional file 3: Table S3). Chr22 had two clusters while Chr12 had only one. Chr06-cluster-1 had 5 QTL, 4 of them stable, explaining 7.8-14.4% of the phenotypic variance. Chr21-cluster-1 had 4 stable QTL for FL, FE, FS and FM explaining 6.9-18.1% of the phenotypic variance. Stability among three environments and significant correlation of fiber quality traits make this QTL cluster particularly worthy of further studies. Chr03-cluster-1 and Chr05-cluster-1 each had 3 QTL, but only one stable QTL. Chr06-cluster-1 had four stable QTL (qFL06.1q, FE06.1, qFS06.1, qFU06.1). Chr07-cluster-1 included 4 QTL for FE, FL, FM, FS explaining 5.9-12.7% of the phenotypic variance, two of which (qFM07.1, qFS07.1) were stable. Chr09-cluster-1 had 3 QTL (qFE09.1, qFM09.2, qFS09.1), all stable, explaining 7.7% of phenotypic variance. Chr10-cluster-1 had 4 QTL, two of which (qFE10.1, qFS10.1) were stable. Ch11-cluster-1 and Chr12-cluster-1 each had 3 QTL, two of which were stable, explaining 9.4% and 11% of the phenotypic variance respectively. Ch16-cluster-1 had 3 QTL (qFE16.1, qFL16.1, qFS16.2), all stable, explaining 8.3% of the phenotypic variance. There were 4 QTL in Ch.17-cluster-1, only one being stable. Ch22-cluster-1 had 5 QTL, but only two were stable. Chr22-cluster-2 had 4 QTL but none were stable. Chr25-cluster-1 had three QTL, two (qFE25.1, qFL25.1) found across all three environments.

**Table 6:** QTL Clusters for fiber quality traits identified across three environments in the Yumian 1 × CA3084 RIL population

Clusters	QTL in each Cluster^a^	Flanking Markers^b^
Chr03-cluster-1	qFL03.1, qFM03.1, qFS03.1^#^	Marker1411- Marker1449
Chr05-cluster-1	qFL05.1, qFM05.1^#^, qFS05.1	Marker2819-Marker2878
Chr06-cluster-1	qFL06.1^##^, qFM06.1, qFE06.1^#^, qFS06.1^#^, qFU06.1^#^	Marker3657-Marker4031
Chr07-cluster-1	qFE07.1, qFL07.1, qFM07.1^##^, qFS07.1^#^	Marker5288-Marker5409
Chr09-cluster-1	qFE09.1^#^, qFM09.2^#^, qFS09.1^#^	Marker10981-Marker11225
Chr10-cluster-1	qFE10.1^#^, qFL10.1, qFM10.1, qFS10.1^#^	Marker12628-Marker12647
Chr11-cluster-1	qFE11.1^#^, qFM11.1^#^, qFS11.1	Marker12710-Marker12871
Chr12-cluster-1	qFM12.1^##^, qFS12.1, qFU12.1^##^	Marker13500-Marker13741
Chr16-cluster-1	qFE16.1^#^, qFL16.1^##^, qFS16.2^#^	Marker18792-Marker18810
Chr17-cluster-1	qFL17.2, qFM17.1, qFS17.1, qFU17.1^#^	Marker16240-Marker16299
Chr21-cluster-1	qFE21.1^#^, qFL21.1^#^, qFM21.1^##^, qFS21.1^#^	Marker21719-Marker21805
Chr22-cluster-1	qFE22.1, qFL22.1^#^, qFM22.1, qFS22.1^#^, qFU22.1	Marker16371-Marker16390
Chr22-cluster-2	qFE22.2, qFL22.2, qFS22.2, qFU22.2	Marker16505-Marker16721
Chr25-cluster-1	qFE25.1^#^, qFL25.1^#^, qFS25.1	Marker17787-Marker17823

^#^indicates stable QTL identified across two environments. ^##^indicate the stable QTL identified across three environments

^a^FL: fiber length, FU: fiber uniformity, FS: fiber strength, FE: fiber elongation, FM: fiber micronaire.

^b^Flanking markers contained a larger region consisting all QTL in a giving cluster.

## Discussion

### Prominent features of the method SLAF-seq

Specific locus amplified fragment sequencing (SLAF-seq) is an efficient and cost effective tool that uses high-throughput sequencing for genotyping [18], and has been applied in many plant species for developing high density genetic maps and mapping QTL [19–23]. As compared to other high throughput genotyping technologies, such as GBS and RAD-seq, SLAF-seq provides a reliable and cost effective approach for QTL mapping [24]. High quality DNA is required for RAD-seq, with modified protocols necessary for low quality DNA [26, 27]. Shared restriction sites among genetically divergent clades may be lost progressively due to sequence polymorphism [26]. GBS is less complicated and more cost effective than RAD-seq [28]. Although GBS and SLAF-seq share some basic principles, some features distinguish SLAF-seq from GBS, i.e. 1) pre-experiment endonuclease digestion scheme development through *in silico* analysis of available sequencing database; 2) ability to create markers evenly distributed over a genome; 3) high quality markers due to stable sequence depth of each sample; 4) high density genetic map development by exploiting abundant SNPs.

In previous studies, usually one or a few common restriction endonucleases were used for genome digestion. The genome specificity was often ignored, which might lead to uneven distribution of the selected fragments in the whole genome. The SLAF-seq strategy remedies this defect [18, 23, 24]. Furthermore, compared with the highest-density SSR genetic map of an Upland cotton intraspecific population [29], the present map also showed some advantages due to prominent features of SLAF-seq. For example, the total number of markers in the present map is far more than the highest-density SSR map, and marker distribution in the present map is more uniform.

### Genetic map

In previous studies, most cotton genetic maps were based on SSR, AFLP or RFLP. However, it is very difficult to construct a high density genetic map covering the genome sufficiently due to the low polymorphism rate of SSR and other markers which exploit length polymorphism [30–32]. Therefore, SNP markers are a better choice to construct high resolution maps due to their high abundance and even distribution. In the present study, we constructed a high density genetic map which harbored 6254 SNP markers with only 8 gaps larger than 10cM. The distribution of mapped SNP markers was not random throughout the genome. This uneven distribution of markers may be due to the multi-step screening of SLAF markers or selective sweeps during domestication. Good collinearity between physical and genetic maps and higher density suggests that this map is high-quality for QTL mapping.

### Segregation Distortion

Segregation distortion is widely observed in plant species [33]. Many studies have reported some factors influencing this phenomenon, such as species, population type and marker type [34–36]. Some studies also suggest the genetic effect as the main factor controlling segregation distortion. Segregation distortion in the present study largely favored Yumian 1 alleles, in accordance with our previous studies [29, 31, 35, 37, 38] which showed that Yumian 1 plays a significant role in segregation distortion due to its complex genetic background. Previous studies suggest that segregation distortion will not affect QTL detection if the distorted marker is not significantly linked to the QTL. Large populations can help to decrease the effect of segregation distortion. Fang et al. [39] fine-mapped a QTL (qFS07.1) located in an SDR , illustrating that segregation distortion does not significantly impact QTL position. In some cases segregation distortion may contribute to higher genetic variation, which in turn benefits QTL detection [40].

### QTL identification

Significant differences among the RIL population showed that the parents had diverse alleles for many fiber quality traits. Among the total of 95 detected QTL, Yumian 1 contributed 46 favorable alleles while CA3084 contributed 49. Each of the parents contributed equally for many traits. QTL diversity among phenotypically similar parent is consistent with previous reports [29, 41–43]. Many QTL for different traits showed overlapping regions, which suggests that these QTL may represent genes with pleiotropic effects contributing to the fiber development network and may contribute to the significant correlations among different fiber quality traits [25, 35, 41, 44–46]. A total of 53 detected QTL occurred in 14 clusters on 13 chromosomes. Yumian 1 contributed to all QTLs in Chr.12-cluster-1 and Chr.16-cluster-1. While CA3084 contributed to the all QTL in Chr.25-cluster-1. In rest of the QTL clusters Yumian 1 was the main contributor. Fiber micronaire alleles in all QTL clusters were contributed by CA3084 except for Chr.11-cluster-1 and Chr.12-cluster-1. This was the proof of expected segregation of the trait because of significant difference of this trait of this trait between the parents [32, 46, 47]. Some clusters had notable numbers of stable QTL. For example, Chr21-cluster-1 had 4 QTL mapped at approximately the same location. All QTL in this cluster were stable, explaining 6.9-18.1% of total phenotypic variance. These QTL may be high priorities for fine mapping studies and candidate gene identification.

Concentration of the most of QTL in 14 clusters confirmed the significant positive correlations among fiber quality traits except for fiber micronaire. Similar results were reported in previous studies [29, 35, 47–50]. This can be the result of presences of multiple genes in certain genomic region or pleiotropic effects of certain genes [25].

The fiber micronaire QTL in the clusters had no consistent correlation in additive effects, hence, showing insignificant phenotypic correlation between micronaire and other traits in the current study. Similar results have been reported in previous study using Yumian 1 as a parent [47].

### Stable and common QTL

Environment plays an important role in phenotypic variation of fiber quality traits. Dissection of variability according to environmental and genetic factors enables us to identify ‘stable’ QTL, i.e., which tend to maintain their effects across multiple environments and are high-priority candidates for further studies. In the present study, 55 of 95 detected QTL were found in two or more environments.

We compared the detected QTL with CottonQTLdb (release 2.2, February 01, 2017) [4, 25] based on the physical position of the nearest DNA markers. In total, 59 QTL (Additional file 4: Table S4) corresponded closely with the physical positions on the TM-1 reference genome of previously reported QTL [13], while the rest were newly found in this study. We also compared our QTL results with recently published GWAS related to cotton fiber traits [51–53], comparing the physical position of our SNP markers linked with QTL to those of GWAS-identified markers (Additional file 4: Table S4). Only 5 QTL corresponded closely with the physical positions of GWAS markers. These common and stable QTL could be priorities for fine mapping, candidate gene identification and MAS to improve cotton fiber quality. Unstable QTL can be the result of imprecise coarse mapping, or unusual environmental conditions.

## Conclusion

In this study, we constructed a high-density genetic map by using SLAF-seq, which covers 3141.7 cM of recombinational length. The average distance between markers was 0.5 cM. A total of 13 QTL clusters containing 29 stable QTL were identified. Some of the stable QTL (qFL06.1, qFM07.1^,^ qFL16.1, qFL21.1) could be valuable for fine mapping and candidate gene identification and can be used in further breeding programs.

## Methods

### Mapping Population

*G. hirsutum* cultivars Yumian 1 and CA3084 were crossed to produce a segregating population at Southwest University, Chongqing, China, in the summer of 2010 [38]. Yumian 1 is a Chinese cultivar with high fiber strength, bred by our lab [35] while CA3084 is an American breeding line with high fiber quality (provided by Dr. John R. Gannaway at Texas A & M University Research Experimental Center, Lubbock, TX). Single-seed descent was used from the F_2:3_ to F_2:7_ generations to produce a RIL population with 180 lines. The RIL population was grown in three different environments (2015, 2016 in Chongqing, 2016 in Hainan).

### Phenotypic data analysis

Mature bolls of the two parents and recombinant inbred lines were harvested manually and fiber quality was measured with HVI900 instruments at the Supervision Inspection and Testing Cotton Quality Center, Anyang, China. The fiber quality traits measured in our study mainly included upper half mean length (FL, mm), fiber strength (FS, cN/tex), fiber elongation (FE), fiber micronaire (FM) and fiber uniformity (FU). These data were analyzed by SPSS 17.0 (SPSS Inc. Released 2008. SPSS Statistics for Windows, Version 17.0. Chicago: SPSS Inc.).

### DNA extractions, SLAF library construction and high-throughput sequencing

In the summer of 2015, the leaves of parents and 87 lines were sampled for DNA extraction. Total genomic DNA was extracted according to a modified CTAB method [54]. To assess the number of markers generated by the combination of different endonucleases, an [18, 55] *in silico* analysis was carried out using a *G. hirsutum* reference genome [13]. Based on this analysis, two endonucleases *HaeIII* and *SspI* (New England Biolabs, USA) were selected to digest the genome of mapping population. The SLAF-seq strategy with some modifications was followed for library construction.

### Sequencing data grouping and genotyping

We identified and genotyped SLAF markers according to the procedures described by Sun et al [18] and Jiang et al [55]. Briefly, low-quality reads (< 20e) were filtered out and the remaining high quality reads were sorted to each progeny according to duplex barcode sequences. Then the terminal 5-bp of each high-quality read was trimmed off. Finally, 80 bp paired-end clean reads obtained from the sample were mapped to the *G. hirsutum* genome sequence [13] using BWA software (V 1.5) with default parameters [56]. Sequences mapped on the same position with over 95 % identity were defined as one SLAF locus [55]. SNP loci in each SLAF locus were then detected between parents using the software GATK (V 3.1.1) with default parameters [57]. All polymorphic SLAF loci with a sequence depth of more than 10 fold in parents were genotyped and the individuals were genotyped based on similarity to the parents. Genotype scoring was conducted to approve the genotyping quality by using a Bayesian approach as described [18]. Markers with more than 15% missing data were removed.

### Linkage map construction

SLAF markers were arranged in specific order and genotyping errors were corrected by using the HighMap strategy and SMOOTH algorithm [58, 59]. The k-nearest neighbor algorithm was used to deal with missing genotypes [60]. Then, the genetic linkage map was constructed by Joinmap v4.0 [61]. The genetic map was constructed according to the maximum likelihood method [62]. The Kosambi mapping function was applied to estimate the genetic map distances in centimorgan (cM) [63]. Markers showing significant deviation from Mendelian expectations for segregation (*p* <0.05) were considered distorted. Regions containing more than three adjacent loci which showed significant segregation distortion were denoted ‘segregation distortion region(s)’ (SDR) [64].

### QTL analysis

MapQTL 6.0 [65] was used to identify QTL. A threshold value of log of odds ratio (LOD) ≥2.0 was used to declare suggestive QTL [66]. Positive additive effect means that favorable alleles were contributed by Yumian 1 while negative additive effect means that favorable alleles were contributed by CA3084. QTL identified in two or more environments were considered to be potential stable QTL. MapChart 2.2 [67] was used to graphically represent the genetic map and QTL bars. QTL were named starting with ‘q’, followed by a trait abbreviation and the chromosome number, then by the number of QTL affecting the trait on the same chromosome (e.g., qFE06.1 for the first fiber elongation QTL on chromosome 6). QTL for the same trait across different environments were declared in the same QTL region when their confidence intervals (CI) overlapped.

## Additional files

Additional file 1: **Table S1.** Genome coverage of genetic map (XLSX 11 kb)

Additional file 2: **Table S2.** QTL for fiber quality in RIL population in three environments (XLSX 20 kb)

Additional file 3: **Table S3.** Details of QTL Clusters for fiber quality traits (XLSX 14 kb)

Additional file 4: **Table S4.** Common QTL compared with CottonQTLdb (XLSX 15 kb)

## Abbreviations

Chr: Chromosome
CI: Confidence interval
FE: Fiber elongation
FL: Fiber length
FM: Fiber micronaire
FS: Fiber strength
FU: Fiber uniformity
GBS: Genotyping by sequencing
LOD: Log of Odds
MAS: Marker assisted selection
NGS: Next generation sequencing
PVE: Phenotypic variance explained
QTL: Quantitative trait locus/loci
RAD: Restriction-site associated DNA
RIL: Recombinant inbred line
SDM: Segregation distortion marker
SDR: Segregation distortion region
SLAF-seq: Specific locus amplified fragment sequencing
SNP: Single nucleotide polymorphism
SSR: Simple sequence repeat(s)
